# Automated characterization of patient–ventilator interaction using surface electromyography

**DOI:** 10.1186/s13613-024-01259-5

**Published:** 2024-02-26

**Authors:** Julia Sauer, Jan Graßhoff, Niklas M. Carbon, Willi M. Koch, Steffen Weber-Carstens, Philipp Rostalski

**Affiliations:** 1 Institute for Electrical Engineering in Medicine, Universität zu Lübeck, Ratzeburger Allee 160, Lübeck, 23562 Germany; 2 Fraunhofer IMTE, Fraunhofer Research Institution for Individualized and Cell-Based Medical Engineering, Lübeck, Germany; 3 Department of Anesthesiology and Intensive Care Medicine, Charité-Universitätsmedizin Berlin, corporate member of Freie Universität Berlin and Humboldt Universität zu Berlin, Berlin, Germany; 4 Department of Anesthesiology, Friedrich-Alexander-Universität Erlangen-Nürnberg, Uniklinikum Erlangen, Erlangen, Germany

**Keywords:** Mechanical ventilation, Patient–ventilator asynchrony, Automation, Surface electromyography, Esophageal pressure

## Abstract

**Background:**

Characterizing patient–ventilator interaction in critically ill patients is time-consuming and requires trained staff to evaluate the behavior of the ventilated patient.

**Methods:**

In this study, we recorded surface electromyography ($\textrm{sEMG}$) signals from the diaphragm and intercostal muscles and esophageal pressure ($P_{\textrm{es}}$) in mechanically ventilated patients with ARDS. The sEMG recordings were preprocessed, and two different algorithms (triangle algorithm and adaptive thresholding algorithm) were used to automatically detect inspiratory patient effort. Based on the detected inspirations, major asynchronies (ineffective, auto-, and double triggers and double efforts), delayed and synchronous triggers were computationally classified. Reverse triggers were not considered in this study. Subsequently, asynchrony indices were calculated. For the validation of detected efforts, two experts manually annotated inspiratory patient activity in $P_{\textrm{es}}$, blinded toward each other, the $\textrm{sEMG}$ signals, and the algorithmic results. We also classified patient–ventilator interaction and calculated asynchrony indices with manually detected inspirations in $P_{\textrm{es}}$ as a reference for automated asynchrony classification and asynchrony index calculation.

**Results:**

Spontaneous breathing activity was recognized in 22 out of the 36 patients included in the study. Evaluation of the accuracy of the algorithms using 3057 inspiratory efforts in $P_{\textrm{es}}$ demonstrated reliable detection performance for both methods. Across all datasets, we found a high sensitivity (triangle algorithm/adaptive thresholding algorithm: 0.93/0.97) and a high positive predictive value (0.94/0.89) against expert annotations in $P_{\textrm{es}}$. The average delay of automatically detected inspiratory onset to the $P_{\textrm{es}}$ reference was $-$79 ms/29 ms for the two algorithms. Our findings also indicate that automatic asynchrony index prediction is reliable. For both algorithms, we found the same deviation of $0.06\pm 0.13$ to the $P_{\textrm{es}}$-based reference.

**Conclusions:**

Our study demonstrates the feasibility of automating the quantification of patient–ventilator asynchrony in critically ill patients using noninvasive sEMG. This may facilitate more frequent diagnosis of asynchrony and support improving patient–ventilator interaction.

**Supplementary Information:**

The online version contains supplementary material available at 10.1186/s13613-024-01259-5.

## Introduction

Patient–ventilator asynchrony is caused by a temporal mismatch between spontaneous patient efforts and ventilatory assistance regarding triggering and cycling off. It is associated with less successful weaning [1], longer duration of mechanical ventilation [2] and higher mortality [3]. Furthermore, asynchrony is believed to cause patient discomfort [4]. Several studies have investigated the prevalence of asynchrony in patient–ventilator interaction during different ventilatory settings such as pressure support ventilation with intubation [1, 2, 5–7], noninvasive ventilation (NIV) [4, 6] and home-NIV [8, 9].

Different types of asynchrony have been observed in mechanically ventilated patients, such as ineffective, auto-, and double triggers that disrupt the normal breathing rhythm. Beyond that, assisted breaths can be affected by minor asynchrony events, sometimes referred to as dyssynchronies, which occur when triggering or cycling is too early or too late [10]. Identifying these classes relies on recognizing specific patterns in the airway pressure and flow curves [6, 10–12] or segmenting the patient’s inspiratory efforts [13, 14].

A large number of publications deal with the precise quantification of asynchrony—different asynchrony indices have been proposed, among others, the ratio of asynchronous breaths to all breaths [2] and the NeuroSync index [13]. In any case, a reliable representation of the patient’s spontaneous breathing activity is required to characterize patient–ventilator interaction accurately. Table 1 summarizes how previous studies have approached this issue. Several authors have attempted to detect and classify asynchronous events in the airway pressure and flow curves both manually [2, 10, 11] and automatically [6, 12]. Furthermore, a growing body of literature exists on patient–ventilator asynchrony detection using esophageal pressure $P_{\textrm{es}}$ [1, 5, 6, 10], invasively measured diaphragm electromyogram $\mathrm {EA_{di}}$ [5, 7, 11, 13] or noninvasive surface electromyography ($\textrm{sEMG}$) [4, 8, 9, 14, 15].

**Table 1:** Selection of previous works on detecting and evaluating patient–ventilator asynchrony

Publication	Signals (reference)	Analysis
Chao et al. (1997) [1]	$\dot{V}$, $P_{\textrm{aw}}$, $P_{\textrm{es}}$	Manual classification of ineffective efforts
Parthasarathy et al. (2000) [5]	$\dot{V}$, $P_{\textrm{es}}$, $P_{\textrm{di}}$, ($\mathrm {EA_{di}}$)	Manual segmentation of patient effort
Thille et al. (2006) [2]	$\dot{V}$, $P_{\textrm{aw}}$	Manual classification of trigger asynchrony events
Mulqueeny et al. (2007) [6]	$\dot{V}$, $P_{\textrm{aw}}$, ($P_{\textrm{di}}$)	Automated detection of ineffective and double triggering
Vignaux et al. (2009) [4]	$\dot{V}$, $P_{\textrm{aw}}$, $\mathrm {sEMG_{di}}$	Manual classification of asynchrony events
Piquilloud et al. (2011) [7]	$\dot{V}$, $P_{\textrm{aw}}$, $\mathrm {EA_{di}}$	Manual classification of asynchrony events
Colombo et al. (2011) [11]	$\dot{V}$, $P_{\textrm{aw}}$, ($\mathrm {EA_{di}}$)	Manual classification of asynchrony events
Carteaux et al. (2012) [15]	$\dot{V}$, $P_{\textrm{aw}}$, $\mathrm {sEMG_{di}}$, $\textrm{sEMG}$ of neck muscles	Manual classification of asynchrony events
Sinderby et al. (2013) [13]	$P_{\textrm{aw}}$, $\mathrm {EA_{di}}$	Manual and automated segmentation of inspirations and classification of minor and major asynchrony events
Ramsay et al. (2015) [8]	$P_{\textrm{aw}}$, $\mathrm {sEMG_{ic}}$, RIP of chest wall and abdomen	Manual classification of asynchrony events
Garcia-Castellote et al. (2017) [16]	$\dot{V}$, $P_{\textrm{di}}$, invasive $\mathrm {EMG_{di}}$, $\mathrm {L_{di}}$	Manual and automated segmentation of patient effort
Duiverman et al. (2017) [9]	$P_{\textrm{aw}}$, $\mathrm {sEMG_{di}}$, $\mathrm {sEMG_{ic}}$, $\textrm{sEMG}$ of scalene muscles	Manual classification asynchrony events
Estrada et al. (2018) [18]	$\dot{V}$, $\mathrm {sEMG_{di}}$	Automated segmentation of patient effort
Koopman et al. (2018) [14]	$P_{\textrm{aw}}$, $\mathrm {sEMG_{di}}$	Manual and automated segmentation of patient effort
Mojoli et al. (2022) [10]	$\dot{V}$, $P_{\textrm{aw}}$, ($P_{\textrm{es}}$)	Manual segmentation of patient effort and classification of asynchrony events
Bakkes et al. (2023) [12]	$\dot{V}$, $P_{\textrm{aw}}$, ($P_{\textrm{es}}$)	Automated segmentation of patient effort and classification of asynchrony events
Telias et al. (2023) [17]	$P_{\textrm{es}}$, $P_{\textrm{aw}}$, $\dot{V}$	Automated segmentation of patient effort and classification of asynchrony events

In recent studies, asynchrony classification and evaluation are typically performed manually. However, manual analysis of waveforms in clinical routine is time-consuming and requires trained staff. Therefore, detecting the patient’s inspiratory activity in $\mathrm {EA_{di}}$ has already been successfully implemented in an automated fashion, which is made possible by its high signal-to-noise ratio. To this end, Sinderby et al. [13] proposed to use a constant threshold of 0.5 µV. When the threshold is exceeded, an inspiratory onset is detected; a drop to less than 70 % of the inspiratory maximum indicates the activity end. The automated analysis of esophageal pressure is complicated by cardiac artifacts and the chest wall recoil pressure. The first algorithmic approaches for patient effort segmentation in $P_{\textrm{es}}$ have been described by Castellote et al. [16]. Recently, Telias et al. [17] have automatically detected asynchrony based on esophageal pressure.

Respiratory sEMG represents a noninvasive alternative to $\mathrm {EA_{di}}$ and $P_{\textrm{es}}$ for detecting patient efforts. Previous studies investigated sEMG recordings of the diaphragm or auxiliary muscles in manual asynchrony detection [4, 8, 9, 15]. An automated method for detecting sEMG would provide valuable assistance for the mentioned reasons. Unfortunately, there is currently no established approach for this. Applying a constant threshold for the onset detection seems unreasonable for $\textrm{sEMG}$ signals. Compared to $\mathrm {EA_{di}}$, the signal-to-noise ratio is lower, and the noise level varies over time. For this reason, there is ongoing research on automated onset detection in $\textrm{sEMG}$ signals with more sophisticated algorithms. For example, Estrada et al. [18] presented a dynamic thresholding approach. Also, an $\textrm{sEMG}$-based implementation of the NeuroSync index [13] has already been proposed and implemented by Koopman et al. [14]; however, the obtained results were not validated via comparison against an independent reference in their study.

This study aims to investigate and validate an automated characterization of patient–ventilator interaction using $\textrm{sEMG}$, potentially promoting broader use of this noninvasive monitoring tool.

## Methods

### Clinical data

We included adult patients with ARDS, who were admitted to one of the participating intensive care units (ICU) at Charité – Universitätsmedizin Berlin, the tertiary care university hospital in Berlin, Germany. Patients were excluded if they had a preexisting lung condition that would significantly change lung mechanics (e.g., COPD GOLD 3-4, pulmonary fibrosis, cystic fibrosis), unfavorable prognosis with expected death within the following days, or pregnant or breastfeeding patients. Consent was obtained from the patient proxy or legal representative and from the patients themselves as soon as they could give consent. The study was approved by the local ethics committee (EA4/005/19) and registered at the German Clinical Trials Register (DRKS00017138).

We defined three treatment phases within our observatory trial as a model of the clinical pathway of ARDS treatment. The first measurement was conducted within the first 48 h after ICU admission. During this phase, ventilation with high PEEP and low tidal volumes was achieved by mandatory ventilation. The second recording was scheduled during the reduction of sedation, defined as the first day with goal $\text {RASS}>-2$, which was set by treating physicians independently from the research team. If no patient respiratory activity was detected in ventilator ($P_{\textrm{aw}}$ and flow) or esophageal pressure curves, this measurement was repeated the following day. The third measurement was performed during the first spontaneous breathing trial, indicated by the treating physician. Again, if no respiratory activity was detected, this measurement was repeated the following day. During all three treatment phases, patients were ventilated with pressure controlled ventilation with the possibility to trigger the ventilator or pressure support ventilation with controlled breaths as a fallback. Throughout the study, we used only flow triggering. All ventilator parameters were set by the treating physicians.

We obtained airway pressure, airflow, and esophageal pressure measurements for each recording with 100 Hz sampling rate. Airway pressure and flow were accessed via the Dräger Medibus interface. Esophageal pressure was obtained via the NutriVent catheter after ensuring the correct catheter position and balloon filling with an occlusion maneuver [19]. We recorded surface EMG with five electrodes at a sampling rate of 1000 Hz using an sEMG amplifier prototype provided by Dräger (Drägerwerk AG & Co. KGaA, Lübeck, Germany). Electrodes for the parasternal channel were placed left and right parasternally in the second intercostal space. For the diaphragm channel electrodes were placed on the costal margin at the left and right medioclavicular line. The ground electrode was placed at the center of the sternum. All measurements were performed in a semirecumbent position with a head elevation of 30 ^∘^.

### Data preprocessing

Cardiogenic pressure artifacts in the esophageal pressure $P_{\textrm{es}}$ were suppressed [20] to recognize inspiratory onsets more accurately. The detection of the end of inspiration was done in the derived pressure $P_{\textrm{mus}}$. For this purpose, the chest wall elastance was determined, and $P_{\textrm{es}}$ was corrected for volume-dependent elastic recoil of the chest wall. Refer to Additional file 1 for further details.

The $\textrm{sEMG}$ signals were denoised by filtering powerline interference and removing electrical cardiac artifacts [21]. To obtain the $\textrm{sEMG}$ envelope, the denoised signal was smoothed using a root-mean-square filter with a 250 ms window. To compensate for the total signal processing and neuromechanical delay prior to further analyses, we parameterized the filter lag to minimize the delay between electrical muscle activity and $P_{\textrm{mus}}$ across our cohort. Details regarding the data preprocessing can be found in Additional file 1.

### Automated sEMG segmentation

Two different algorithms were employed for detecting the start of inspiration in the selected $\textrm{sEMG}$ envelope. The first algorithm (triangle algorithm) was designed for maximum robustness and highly accurate detection of the exact onset of electrical activity. In contrast, the second algorithm (adaptive thresholding algorithm) was tailored to be highly sensitive to small patient activities. Detailed descriptions of $\textrm{sEMG}$ detection algorithms are provided in Additional file 1.

**Algorithm 1:**
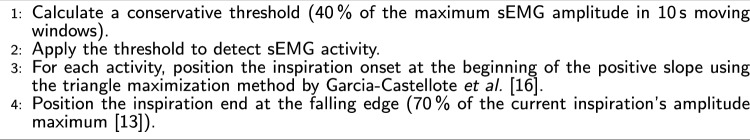
Triangle algorithm for maximum robustness and highly accurate detection of the exact onset of electrical activity.

**Algorithm 2:**
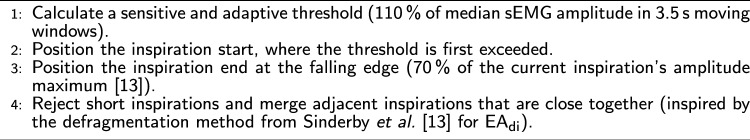
Adaptive thresholding algorithm designed for high sensitivity to minimal patient activities.

Prior to the segmentation, both $\textrm{sEMG}$ channels were analyzed computationally to check if patient activity was present in the signal, and the signal quality was evaluated based on the signal-to-noise ratio. Channels that showed patient activity and sufficient signal quality were automatically segmented by both algorithms. If no channel of a recording was valid, no $\textrm{sEMG}$ segmentation was performed. In case both channels were found to be usable for segmentation, for each breath, the earlier of the two detected onsets was used as the start of electrical activity. Details are given in Additional files 1 and 2.

### Automated characterization of patient–ventilator interaction

**Fig. 1:**
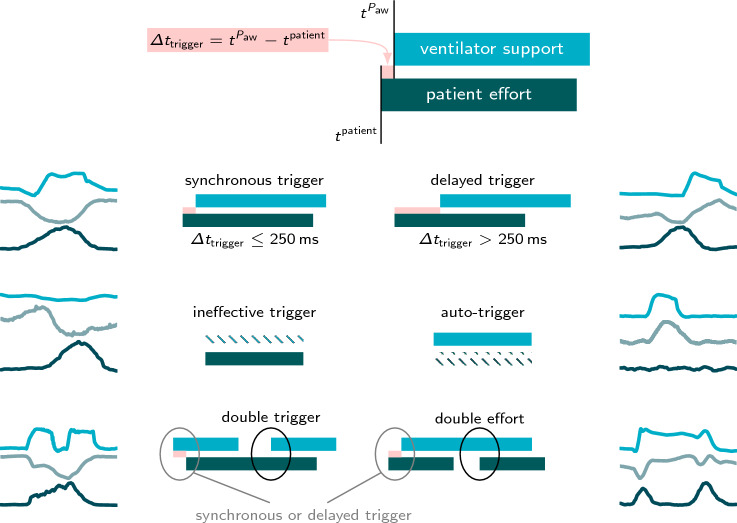
Definitions of considered patient–ventilator interactions based on the segmentation of the ventilator support (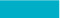) in $$P_{\textrm{aw}}$$ (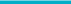) and the patient effort (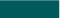) in $$\textrm{sEMG}$$ (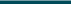). Reference segmentations of the patient effort are based on $P_{\textrm{es}}$ (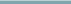). For distinguishing synchronous and delayed triggers, a threshold of ${250\,\textrm{ms}}$ is applied to the derived trigger delay $\Delta t_{\textrm{trigger}}$ (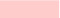) [10]. The absence of ventilator support or patient activity corresponds to an ineffective or auto-trigger, respectively. If a single patient effort overlaps with two ventilator supports, the first is classified according to the trigger delay, whereas the second is called a double trigger. Similarly, if two patient efforts overlap with a single ventilator support, the second is a double effort

The interaction of patient and ventilator was characterized computationally by comparing the detected inspiratory effort against the timing of ventilatory support. As no internal trigger signal of the ventilator was available, trigger and cycling-off times were obtained from automatically segmented airway pressure $P_{\textrm{aw}}$. The mechanical breath was assumed to start with the first sample of the positive ramp in the airway pressure signal and to end with the first sample of the falling edge. We employed six different classes of patient–ventilator interaction, visualized in Fig. 1. For overlapping segments, we calculated the trigger delay $\Delta t_{\textrm{trigger}}= t^{P_{\textrm{aw}}} - t^{\textrm{patient}}$ with the beginning of the ventilator support $t^{P_{\textrm{aw}}}$ and the onset of patient activity $t^{\textrm{patient}}$. If a single patient effort and single ventilator support overlapped and the trigger delay was $\Delta t_{\textrm{trigger}}\le {250\,\mathrm{\text {m}\text {s}}}$, the breath was classified as synchronous, which is based on the definitions given by Mojoli et al. [10]. Under the same conditions, but with $\Delta t_{\textrm{trigger}}> {250\,\mathrm{\text {m}\text {s}}}$, the trigger was considered to be delayed. Breaths were classified as ineffective and auto-triggers when a patient’s effort did not overlap with any ventilator support or vice versa. If a single patient effort overlapped with two ventilator supports, the first was classified according to its trigger delay, and the second was classified as a double trigger. We also observed a similar constellation, where two patient efforts intersected with a single ventilator support, which we referred to as a double effort [22].

The asynchrony index is defined similarly to Mojoli et al. [10] as the number of major asynchronous events (ineffective, auto- and double trigger, and double efforts) divided by the total number of breaths.

### Expert reference

As a first step toward creating a reference for the timing of spontaneous breathing activity, onsets of spontaneous patient efforts were annotated. Independent of each other, two experts labeled the onset of negative deflection in the filtered $P_{\textrm{es}}$ signal. $P_{\textrm{aw}}$ and flow curves were provided simultaneously, but the experts were blinded toward each other and to the sEMG signals. The annotated onset times by both experts were averaged on a breath-by-breath basis, provided the difference between their annotations was no more than 250 ms. The remaining annotations were considered invalid. For each annotated patient effort, the end of inspiration was computationally detected in the calculated $P_{\textrm{mus}}$ waveform, likewise by identifying the point at which $P_{\textrm{mus}}$ fell below 70 % of its maximum value.

### Data analysis

#### Detection evaluation

Each inspiratory effort detected in the sEMG was evaluated as a true positive if it overlapped with the expert reference segmentation. If it did not overlap with the reference, it was a false positive, and all remaining breaths detected by the experts in $P_{\textrm{es}}$ were false negatives. As performance metrics, we determined the sensitivity and the positive predictive value.

#### Classification evaluation

The aforementioned performance metrics (sensitivity and positive predictive value) were calculated individually in each patient–ventilator interaction class by considering all other classes as negative labels. We also calculated the specificity of algorithms with respect to each class. Finally, aggregated performance scores were determined by averaging the sensitivity, positive predictive value, and specificity across all classes. Here, two variants were used, one using the arithmetic mean and one using the weighted mean (where weights correspond to the number of elements in each class).

#### Statistics and deviation analysis

To account for varying numbers of breaths in different recordings, performance measures were calculated for each recording separately. We used the Wilcoxon rank-sum test to compare the median values of two distributions. Levene’s test was used to test for differences in variances of two distributions. The deviation between detected patient effort onsets, trigger delays, and asynchrony index estimates across all patients is expressed as mean ± standard deviation. Differences were analyzed via the limits of agreement method by Bland and Altman [23]. As there were different numbers of breaths in each patient, we used a variant of the original method that accounts for repeated measurements; refer to [24] for details.

## Results

**Fig. 2:**
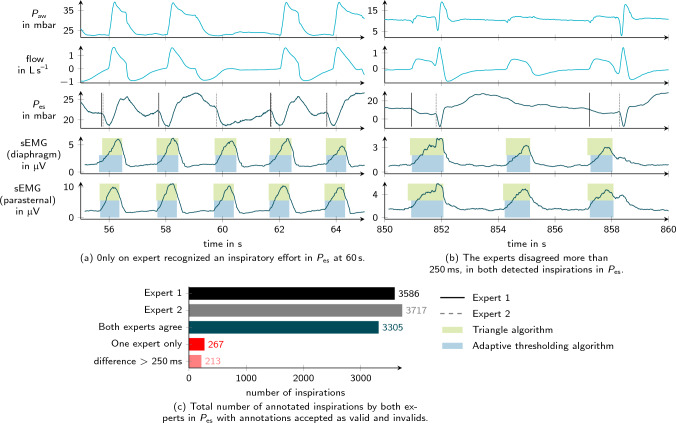
Expert reference in $P_{\textrm{es}}$. **a** and **b** show two examples from two different patients, where the experts disagreed on whether patient activity occurred (**a**) and on the exact onset of activity (**b**). In both examples, the invalid annotations were not taken into account in further algorithm validation. For comparison, the sEMG curves and the results of the algorithms are also plotted in each case. However, the experts were blinded to these while evaluating $P_{\textrm{es}}$. c provides an overview of the total number of annotated breaths in $P_{\textrm{es}}$, including agreements and disagreements

**Fig. 3:**
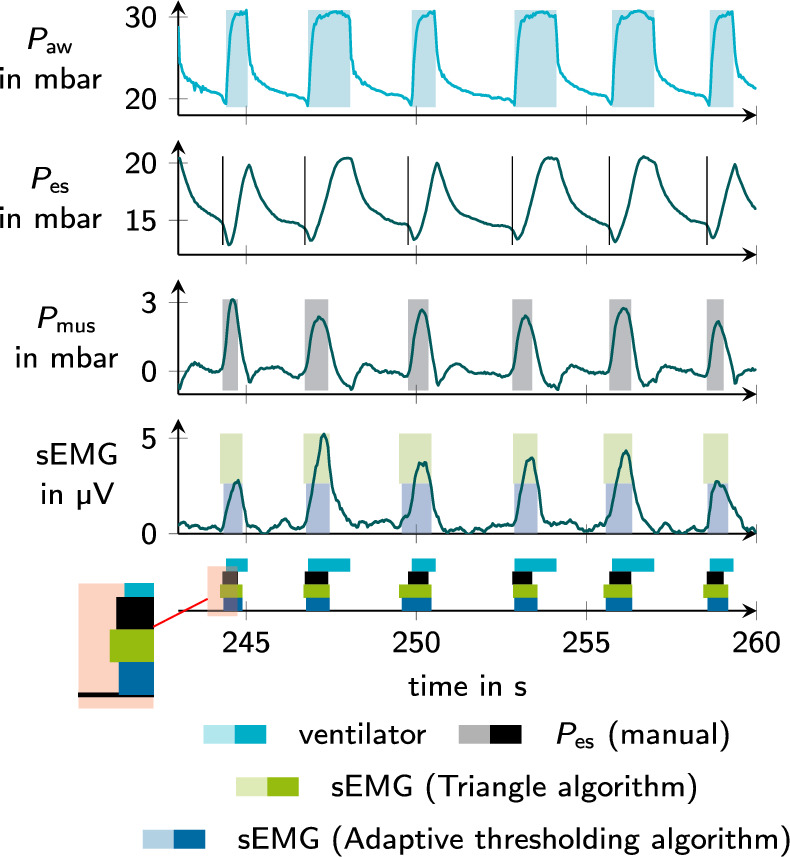
Waveforms and segmentation example. From top to bottom, this figure shows the airway pressure $P_{\textrm{aw}}$ with the segmented ventilator support, the esophageal pressure $P_{\textrm{es}}$ after cardiac artifact removal with black lines representing annotated inspiration starts, the muscular pressure $P_{\textrm{mus}}$ derived from $P_{\textrm{es}}$ which is used for finalizing the reference segmentation of patient inspiratory effort, the electrical muscle activity $\textrm{sEMG}$ with automated segmentation results and finally the summary of all segmented inspirations in $P_{\textrm{aw}}$, $P_{\textrm{es}}$ and $\textrm{sEMG}$. Based on the segmentation results, the asynchrony classification and the asynchrony index calculation are performed. During annotation of $P_{\textrm{es}}$ the experts were blinded towards each other, the $\textrm{sEMG}$ signal and the automated segmentation results

A total of 84 recordings were carried out in 36 patients. The patient characteristics are reported in Table 2. On average 6 min (IQR 4 to 8 min) were analyzed per recording. The goal was to evaluate approximately 100 breaths in each recording. The two experts consistently recognized inspiratory activity in 34 $P_{\textrm{es}}$ recordings. For the analysis, only annotations in which the experts agreed were used, which led to a total of 3305 breaths. Two examples of non-matching and thus excluded inspiratory efforts are shown in Fig. 2. A disagreement between experts occurred in 480 (14 %) annotated patient efforts in $P_{\textrm{es}}$. Table 3 provides an overview of the number of annotated inspirations in $P_{\textrm{es}}$ and the detected efforts in both $\textrm{sEMG}$ channels. An illustrative excerpt with all recording channels and detection results is given in Fig 3.

**Table 2:** Characteristics of analyzed patients ($n=36$)

Characteristic	Result Median (IQR)
Age (years)	61.5 (56.5 to 65.25)
Gender	
Male	25
Female	11
Weight (kg)	90 (80 to 104)
BMI (kg m^-2^)	29.2 (26 to 34.7)
SOFA Score on admission	12.5 (10 to 15)
Cause of ARDS	
HAP	6
CAP	14
Aspiration	9
Covid	7

*BMI* body mass index, *HAP* hospital-acquired pneumonia, *CAP* community-acquired pneumonia

**Table 3:** Overview on the number of annotated breaths in $P_{\textrm{es}}$ and detected breaths in $\textrm{sEMG}$

Signal/setting	Inspirations (recordings)		
	(a) All recordings	(b) Detection validation	(c) Asynchrony validation
$P_{\hbox {es}}$	3305 (34)	3057 (32)	2684 (32)
sEMG (costal margin)	2909/3248 (25)	2789/3116 (24)	2410/2728 (24)
sEMG (parasternal)	3652/4143 (33)	3400/3859 (30)	3052/3476 (30)
sEMG (earliest)	3910/4450 (35)	3650/4165 (32)	3198/3693 (32)

In setting (a), the number of annotated or automatically detected breaths in all recordings where patient activity was observed or detected is given. The second setting (b) considers only recordings with valid expert annotations and detected efforts in at least one sEMG channel (with sufficient signal-to-noise ratio). These are used for validating the detection performance of the algorithms against the annotations in $P_{\textrm{es}}$. The last setting (c) uses only recordings with valid expert annotations, detected efforts in $\textrm{sEMG}$, and ventilation modes that allow triggering by the patient. This excludes signal sections during CPAP. In all cells, the number of recordings is given in brackets. For the sEMG signals, the number of detected breaths by both algorithms is given (triangle algorithm/adaptive thresholding algorithm)

### Detection validation against ${P_{\textsf{es}}}$ reference

**Fig. 4:**
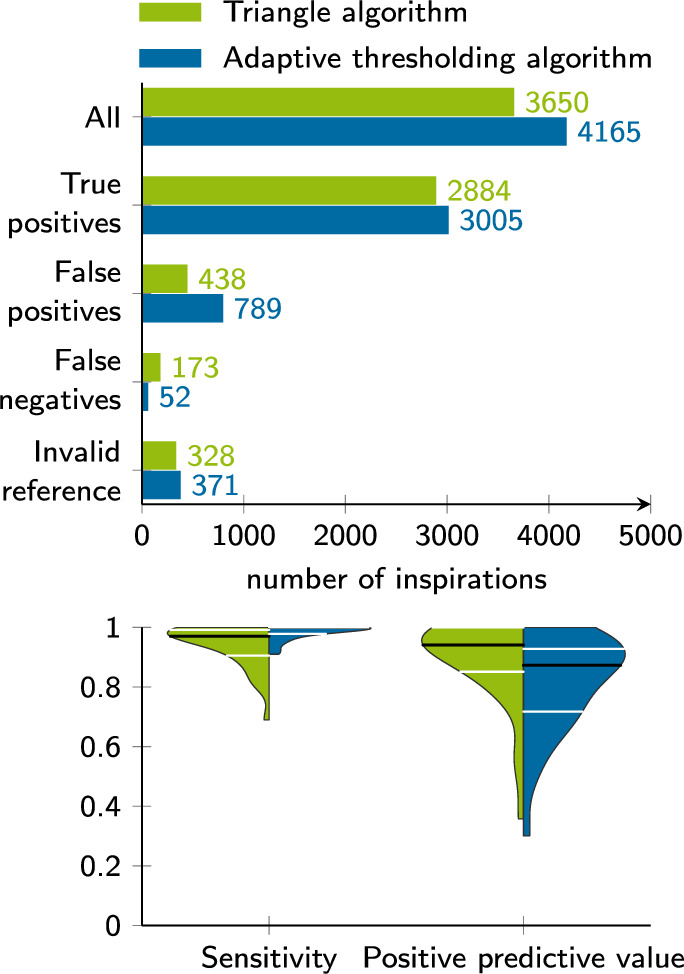
Detection validation against $P_{\textrm{es}}$ reference. The validity of both approaches is examined by comparing automatically detected inspirations in the sEMG and manual annotations in the esophageal pressure. The upper plot overviews the total number of correctly and incorrectly detected patient efforts. The last entry shows how many of the detected breaths were assigned to an uncertain reference where the experts disagreed. The lower plot shows binary metrics to evaluate the detection performance. For sensitivity and positive predictive value, the distribution over patients and recordings is given. Black lines denote the median value, and white lines visualize the interquartile range

**Fig. 5:**
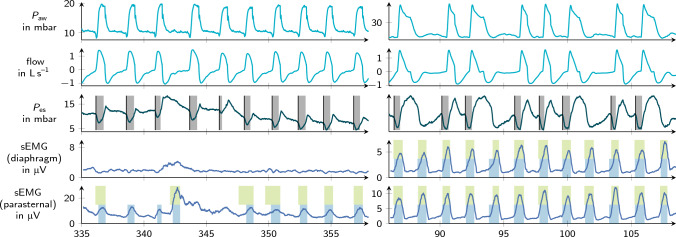
Detection examples that do not match the reference. In the left example, the parasternal sEMG signal is affected by crosstalk or another artifact. This leads to a false positive and multiple false negatives when compared to expert annotations in $P_{\textrm{es}}$. In the diaphragm sEMG, no patient efforts were detected. In the second example, $P_{\textrm{es}}$ and sEMG waveforms disagree, as there are more patient efforts visible in the electrical signal. These are all considered false positives

The total number of patient efforts detected in sEMG and the number of true positives, false positives, and false negatives are reported in the bar chart in Fig. 4. The same figure provides the distribution of sensitivity and positive predictive value across the included datasets and for both algorithms. The median sensitivity was 0.97 (IQR 0.91 to 0.99) for the triangle algorithm and 1.00 (IQR 0.98 to 1.00) for the adaptive threshold algorithm. The positive predictive value results were 0.94 (IQR 0.85 to 1.00) and 0.87 (IQR 0.72 to 0.93) for the triangle and the adaptive thresholding algorithm, respectively. Across all recordings, the triangle algorithm showed fewer false positives and higher positive predictive value ($p=0.011$) but more false negatives and lower sensitivity ($p=0.005$) than the adaptive thresholding algorithm. Examples for breaths, where inspiratory effort could not be detected in sEMG but in $P_{\textrm{es}}$ and vice versa, are presented in Fig. 5. The temporal deviation $t^{\textrm{sEMG}}_{\textrm{automatic}} - t^{P_{\textrm{es}}}_{\textrm{manual}}$ per breath was ($-$0.08 ± 0.27) s for the triangle algorithm and (0.03 ± 0.23) s for the adaptive thresholding algorithm. To better conceive the magnitude of the mean deviation from manual references, refer to the highlighted inspiration in Fig. 3. Here, the deviation of automatic detection in $\textrm{sEMG}$ and manual annotation in $P_{\textrm{es}}$ was very close to the mean deviation across all inspirations.

### Patient–ventilator interaction

**Table 4:** Patient–ventilator interaction classification results

$P_{\hbox {es}}$ reference	Synchronous trigger	Delayed trigger	Auto-trigger	Ineffective trigger	Double trigger	Double effort	Not detected
	*Triangle algorithm*						
Synchronous trigger	1825	328	108	1	11	2	13
Delayed trigger	66	185	2	3	20	0	1
Auto-trigger	149	43	334	0	6	0	1
Ineffective trigger	0	1	0	66	0	0	46
Double trigger	0	0	2	0	37	1	0
Double effort	0	0	0	0	0	4	2
Not detected	14	12	1	182	0	10	0
	*Adaptive thresholding algorithm*						
Synchronous trigger	2080	154	41	1	8	1	3
Delayed trigger	149	104	1	2	18	1	2
Auto-trigger	225	38	268	0	1	0	1
Ineffective trigger	0	2	0	72	0	0	39
Double trigger	0	1	1	0	38	0	0
Double effort	0	0	0	0	0	5	1
Not detected	8	3	1	474	0	19	0

Each row in the table represents the reference events based on manual annotations in $P_{\textrm{es}}$ and each column provides the asynchrony events based on automated $\textrm{sEMG}$ detection

**Fig. 6:**
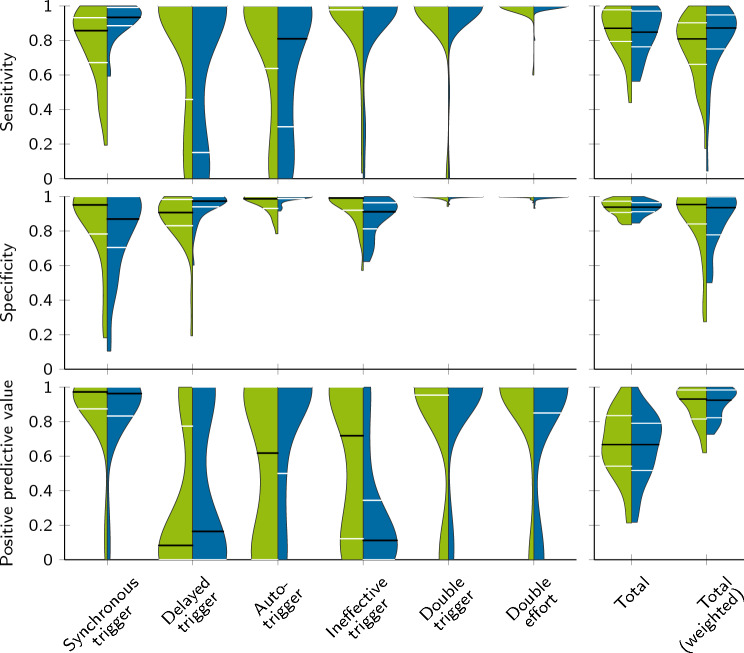
Patient–ventilator interaction classification metrics. For the triangle algorithm (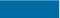) and the adaptive thresholding algorithm (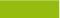), the six classes and 32 recordings, the sensitivity, specificity and positive predictive value are evaluated. This plot visualizes the distribution of the three metrics over the datasets. Black lines indicate the median value and white lines mark the interquartile range. In addition, the results of the different classes are summarized on the right side, where Total denotes the average over all classes and in Total (weighted) considers the class frequency. In this way, the latter represents a reality-oriented outcome

The number of correctly and incorrectly classified breaths from the two algorithms can be seen in Table 4. Figure 6 shows the distribution of sensitivity, specificity, and positive predictive value across all recordings for all asynchrony classes and both algorithms. Both approaches achieve high median sensitivity $\ge 0.81$ and specificity $\ge 0.87$ across all classes. The adaptive thresholding algorithm was less precise in classifying ineffective triggers, whereas the triangle algorithm showed partially incorrect classifications for auto-triggers. Across all classes, we did not find significant differences in the total classification performance between both methods ($p \ge 0.131$ for all classes). In this context, it should be mentioned that double triggers ($n=40$) and double efforts ($n=6$) occurred only rarely, and the significance is therefore limited.

**Fig. 7:**
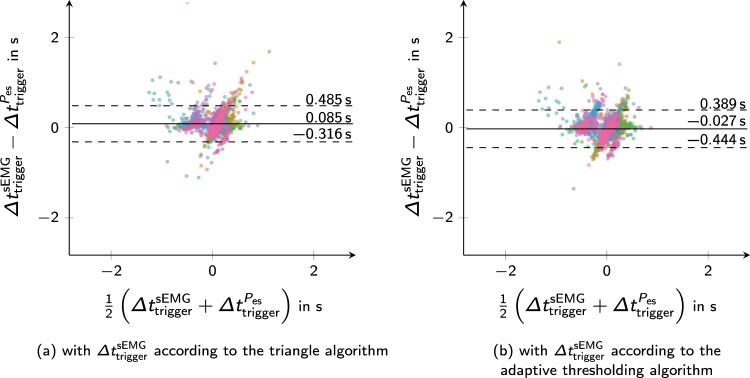
Bland–Altman plots of the trigger delay. The trigger delay $\Delta t_{\textrm{trigger}}$ denotes the difference between the beginning of the ventilator support $t^{P_{\textrm{aw}}}$ and the inspiratory patient effort $t^{\textrm{patient}}$. In both plots, the reference $\Delta t^{P_{\textrm{es}}}_{\textrm{trigger}}$ is built upon the manual annotation of patient efforts in the esophageal pressure $P_{\textrm{es}}$. The left graph compares the trigger delay obtained from the sEMG segmentation results by the triangle algorithm and the second plot shows the results corresponding to the adaptive thresholding algorithm. Each sample in both plots denotes one synchronous or delayed trigger event. Different patients and recordings are characterized by means of colors. Solid lines represent the mean difference and dashed lines mark the $\pm {1.96\,\textrm{Std}}$ intervals around average

Across all recordings, the median misclassification rate between synchronous and delayed triggers is 12 % (IQR 6 to 26 %) for the triangle algorithm and 6 % (IQR 2 to 11 %) for the adaptive threshold algorithm. This distinction was made using the fixed threshold for the trigger delay (${250\,\mathrm{\text {m}\text {s}}}$). To better understand this analysis, Fig. 7 presents Bland–Altman plots for the trigger delay. As the first algorithm detected inspiratory effort earlier, leading to an average increase of the trigger delay by 79 ms, it tended to classify delayed triggers instead of synchronous ones. In contrast, the adaptive thresholding algorithm showed only a minor average systematic deviation of $-$36 ms with similar scattering ($p=0.211$).

**Fig. 8:**
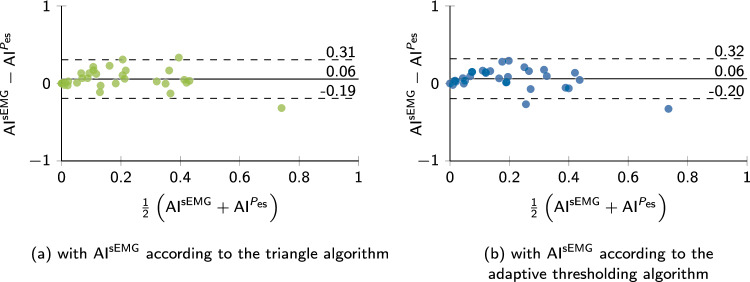
Bland–Altman plots of asynchrony indices. Based on the classification of patient–ventilator interaction, asynchrony indices $\textrm{AI}^{\textrm{sEMG}}$ are determined for the sEMG segmentation results of the triangle algorithm (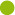) and the adaptive thresholding algorithm (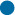). The reference $\textrm{AI}^{P_{\textrm{es}}}$ is calculated based on the expert annotations in $P_{\textrm{es}}$. Solid lines represent the mean difference and dashed lines mark the $\pm {1.96\,\textrm{Std}}$ intervals around average

The evaluated asynchrony indices of each recording are displayed in Bland–Altman plots in Fig. 8. The difference between $\textrm{sEMG}$-based and $P_{\textrm{es}}$-based asynchrony index was $0.06 \pm 0.13$ in both algorithms. Overall, both algorithms performed similarly well, and we could not find a significant difference between algorithms ($p=0.537$).

## Discussion

The present study addresses the automated characterization of patient–ventilator interaction based on $\textrm{sEMG}$ measurements of the diaphragm and the intercostal muscles in a cohort of patients with severe ARDS. Recorded $\textrm{sEMG}$ signals were preprocessed, and inspiratory patient efforts were segmented automatically using two different detection algorithms. Subsequently, patient efforts were related to the ventilator pressure support and assigned to six classes: synchronous, delayed, auto-, ineffective, and double trigger, as well as double efforts. From all the events detected, an asynchrony index was calculated to quantify the level of asynchrony [10]. The automatically detected efforts, assigned classes of patient–ventilator interaction, and asynchrony indices were validated through esophageal pressure $P_{\textrm{es}}$. The results show that there is potential to measure the spontaneous breathing activity noninvasively, to detect onsets of electrical activity in an automated way, and to distinguish different asynchronous events computationally.

Our study makes several significant contributions to the field of patient–ventilator interaction analysis. First, the comparative analysis of two algorithms for sEMG detection allowed us to assess the strengths and limitations of each algorithm. Second, the study population of ARDS patients provides valuable insights on the methods’ applicability to critically ill patients. In addition, we applied our methodology across different treatment phases, ensuring its applicability throughout the course of patient care. Therefore, we employed objective signal criteria such as signal-to-noise ratio to determine the suitability of the sEMG signal for automated analysis in each recording. Furthermore, we computationally characterized the patient–ventilator interaction, which would enable automated sEMG-based asynchrony monitoring. We rigorously validated our findings against expert annotations in $P_{\textrm{es}}$, a well-established reference [6, 10].

Both $\textrm{sEMG}$ detection algorithms showed significant differences in detection accuracy metrics, which can be attributed to their differences in threshold calculation and threshold adaption speed. The adaptive thresholding algorithm missed very few inspirations compared to expert annotations in $P_{\textrm{es}}$, because it quickly adjusts the threshold, allowing the detection of even minor activities. However, results indicate that small disturbances, background noise, or residual cardiac artifacts could lead to falsely detected inspirations. The triangle algorithm yielded a lower false positive rate in detecting inspirations. This could be associated with its threshold calculation, which only considers activities with amplitudes reaching at least 40 % of the maximum amplitude. This approach minimizes the impact of smaller artifacts on the detection performance. On the downside, small activities could be missed more easily. Using the triangle algorithm, most auto-triggers were detected, but there were also many false positives, where small patient efforts were missed. Classification based on the second algorithm showed a reverse tendency. Compared to the reference, more ineffective efforts were captured, but auto-triggers were partially misclassified as synchronous. On average, onsets detected by the triangle algorithm were earlier than manual references, because the algorithm, originally proposed by Garcia-Castellote et al. [16], positions the beginning of activity precisely at the start of the rising edge. In contrast, the adaptive thresholding algorithm was slightly later than the experts because the onset was detected only once the threshold was exceeded. The steeper the slope of the $\textrm{sEMG}$ envelope and the higher the signal amplitude, the closer these two points move together. Low activity amplitudes often result in flat slopes, and thus, more uncertainty in the exact position of the electrical activity onset.

There are several reasons why automatic segmentation might deviate from the annotations in $P_{\textrm{es}}$. On the one hand, captured electrical activity may be crosstalk from other muscles and misinterpreted by algorithms. On the other hand, it is also possible that an inspiratory patient effort was recognized in $\textrm{sEMG}$ but not in $P_{\textrm{es}}$. This could occur when the pressure amplitude is small or the effort is superimposed by the volume component of an assisted breath, particularly in cases of muscle weakness or low effort. These phenomena can hardly be distinguished and would be reflected in a lower positive predictive value in Fig. 4. Due to the lack of a better reference, it is not yet clear if the $\textrm{sEMG}$ detector might, in some cases, be more sensitive to small efforts than $P_{\textrm{es}}$. In other cases, inspirations in the $\textrm{sEMG}$ were superimposed by noise, or the crosstalk increased temporarily. This led to false negatives and affected the detection sensitivity. In Fig. 4, it is evident that some recordings showed good temporal alignment of onsets between $\textrm{sEMG}$ and $P_{\textrm{es}}$, whereas others exhibit systematic biases. These systematic biases cannot be explained through neuromechanical or signal processing delay. One explanation might be that the contributions of different muscles were measured. With $\textrm{sEMG}$, diaphragm and accessory muscles were recorded separately and the latter potentially recruited significantly earlier or later [25]. The systematic differences between the detected onsets in $\textrm{sEMG}$ and the manual reference in $P_{\textrm{es}}$ affected the distinction between synchronous and delayed triggers. A solution might be a modification of the 250 ms threshold to better account for this systematic difference.

Asynchrony indices offer a simplified perspective and condense complex information into a single value. When comparing the automatically determined indices against reference indices, underlying information is lost, and classification may differ yet still result in the same index. This is why we have extensively discussed the two algorithms and their classification performance in previous sections. Overall, both automated approaches provide reliable asynchrony index estimates.

In validating automated characterization of patient–ventilator interaction, consideration should also be given to the shortcomings of the reference. Although esophageal pressure is an established measure for detecting inspiratory patient effort [1, 6, 10, 12], sometimes $P_{\textrm{es}}$ amplitudes were very low and hardly recognizable due to low patient activity, muscle weakness, and the volume component in the signal. Temporarily, the pressure signal might also have been disrupted, e.g., by cardiac artifacts or peristalsis. The preselection of valid reference events showed that the manual recognition and segmentation of patient efforts in the $P_{\textrm{es}}$ curve were partly unreliable because experts disagreed in 14 % of annotated breaths. Another point of discussion is recognizing the end of the inspiratory effort in the esophageal pressure waveform. The manual annotation in $P_{\textrm{es}}$ might be problematic due to the unknown esophageal volume component. We found an acceptable transparent solution for that, estimating $P_{\textrm{mus}}$ from $P_{\textrm{es}}$ by removing the volume component and applying Sinderby’s rule to the $P_{\textrm{mus}}$-waveform (70 % amplitude on the falling edge). Finally, this study does not distinguish between auto-triggers and controlled breaths triggered as a fallback. Consequently, there could be a few controlled breaths among the detected auto-triggers.

The study also revealed that recording the $\textrm{sEMG}$ of intercostal muscles is a valuable extension to the diaphragm, demonstrated by the number of detected efforts in this channel. In 21 out of 43 cases, parasternal $\textrm{sEMG}$ showed a better signal-to-noise ratio and less crosstalk during expiration than the electrode channel above the costal margin measuring the diaphragm. This could be due to the proximity of the abdominal muscles, which might contract during expiration, resulting in crosstalk.

## Conclusion and outlook

The present results show that the detailed characterization of patient–ventilator interaction in a noninvasive and automated manner is similarly accurate to manual and invasive assessments. We found that the two applied algorithms provide reliable asynchrony index results.

In future works, the detection performance might be improved by automated artifact and crosstalk rejection [26]. Furthermore, the complementary properties of the two $\textrm{sEMG}$ detection algorithms suggest the potential for combining the strengths of both approaches, e.g., by averaging over or switching between them.

We hypothesize that when the patient-generated flow is weak or absent due to muscle weakness, $\textrm{sEMG}$ activity would still be measurable, allowing us to detect patient–ventilator asynchrony automatically. In this case, triggering based on noninvasively measured electrical activity could reduce patient–ventilator asynchrony. Interpreting $P_{\textrm{es}}$ curves requires experienced clinical staff. In addition, the positioning of the balloon catheter is prone to error for untrained physicians. Thus, an automated approach based on simple electrical measurements with adhesive electrodes on the thorax could prove to be highly beneficial in clinical routine.

### Supplementary information

**Additional file 1: **Data processing, EMG signal quality criteria and detection algorithms.

**Additional file 2: Figure S1.** Overview on number of recordings per patient. **Table S1.** Number of annotated/detected breaths for each patient and recording. **Figure S2.** Detection validation against *P*_es_ reference. **Table S2.** Numerical results for sEMG detection performance. **Table S3.** Numerical results for PVI classification evaluation. **Figure S3.** Detection validation against sEMG reference.

## Data Availability

The datasets used are available upon reasonable request.

## Abbreviations

$$\textrm{sEMG}$$: Surface electromyography
$$P_{\textrm{es}}$$: Esophageal pressure
$$P_{\textrm{mus}}$$: Muscle pressure
$$P_{\textrm{aw}}$$: Airway pressure
NIV: Noninvasive ventilation
$\mathrm {EA_{di}}$: Invasively measured diaphragm electromyogram
ARDS: Acute respiratory distress syndrome
ICU: Intensive care unit
PEEP: Positive end-expiratory pressure
RASS: Richmond agitation–sedation scale
$t^{\textrm{sEMG}}_{\textrm{automatic}}$: Automatically detected start of inspiration in $\textrm{sEMG}$
$t^{P_{\textrm{es}}}_{\textrm{manual}}$: Manually annotated start of inspiration in $P_{\textrm{es}}$
Std: Standard deviation
$\Delta t_{\textrm{trigger}}$: Trigger delay defined as the temporal difference between ventilator triggering and beginning of patient effort
$\Delta t_{\textrm{trigger}}^{\textrm{sEMG}}$: $\textrm{sEMG}$-based trigger delay
$\Delta t_{\textrm{trigger}}^{P_{\textrm{es}}}$: $P_{\textrm{es}}$-based trigger delay
