# Supplementary material for: Automated characterization of patient–ventilator interaction using surface electromyography
Source: Ann Intensive Care. 2024 Feb 26;14:32. doi: 10.1186/s13613-024-01259-5 (PMC10897101; doi:10.1186/s13613-024-01259-5)
Supplement: Supplementary file 1 — Additional file 1: Data processing, EMG signal quality criteria and detection algorithms. [file 13613_2024_1259_MOESM1_ESM.pdf]

Supplementary information

Automated characterization of patient-ventilator interaction using surface electromyography

Appendix A: Data preprocessing

A.1 Esophageal pressure

Esophageal pressure signals are often strongly contaminated by cardiogenic pressure artifacts, which substantially complicate the underlying respiratory pressure waveform interpretation. Thus, these artifacts were removed prior to further analyses using the template subtraction method described in [1]. Briefly, a template was formed by averaging over many artifacts, and the template was subtracted from P_{es} with each cardiac cycle. The signal filtered in this way enables much more accurate detection of the onset of respiratory effort. The recognition of the end of efforts is still challenging because P_{es} contains a mixture of the pressure P_{mus} generated by respiratory muscles and the volume-dependent elastic recoil of the chest-wall P_{cw}. To correct for P_{cw}, the chest-wall elastance E_{cw} was determined as described in appendix A.2 Finally, the muscle pressure at each instant was calculated via $P_{\text{mus}} = E_{\text{cw}}V - P_{\text{es}}$.

A.2 Chest-wall elastance estimation

For calculating the muscle pressure P_{mus}, the volume-dependent elastic recoil of the chest-wall $P_{\text{CW}} = E_{\text{CW}}V$ and thus, the chest-wall elastance E_{CW} needs to be estimated for each patient and each measurement. We integrated three approaches for computing E_{CW}, with decreasing priority. First, if one or more fully passive breaths (no patient activity) were identified by experts, E_{CW} was estimated by

$$E_{\text{CW}} = \text{argmin} \sum_{t=t_{\text{start}}}^{t_{\text{end}}} \left(E_{\text{CW}}V(t) - P_{\text{es}}(t)\right)^2 \tag{1}$$

where t_{start} and t_{end} are the beginning and end of inspiration defined by the start and end of positive airflow. In case multiple fully passive breaths were recognized, the sum in eq. (1) was extended with these breaths. Second, if experts could not recognize any fully passive breath but could identify one passive point during inspiration and one during expiration in one breathing cycle, the formula

$$E_{\text{CW}} = \frac{P_{\text{es}}(t_1) - P_{\text{es}}(t_2)}{V(t_1) - V(t_2)} \tag{2}$$

was used. Here t_1 and t_2 are the annotated passive points. In case multiple breathing cycles with two passive samples could be identified, eq. (2) was applied to each

pair. Subsequently, E_{CW} was computed by averaging over the results. Third, in case previous approaches to determining E_{CW} could not be applied, a theoretical value was assumed. Therefore, the vital capacity of female patients is calculated by

$$\mathrm{VC}\ [\mathrm{cm}^3] = (21.78 - 0.101 \cdot \mathrm{age}\ [\mathrm{years}]) \cdot \mathrm{height}\ [\mathrm{cm}] \tag{3}$$

and we used

$$\mathrm{VC}\ [\mathrm{cm}^3] = (27.63 - 0.112 \cdot \mathrm{age}\ [\mathrm{years}]) \cdot \mathrm{height}\ [\mathrm{cm}] \tag{4}$$

for male patients [2]. Finally, we computed E_{CW} according to Mauri *et al.* [3] with 4 % of VC.

A.3 Electromyographic signals

Both recorded sEMG channels (costal margin and parasternal) were preprocessed individually. At first, powerline interference was removed using a zero-phase fourth-order Butterworth band-stop filter. Next, cardiac artifacts were detected with the Pan-Tompkins algorithm and suppressed using wavelet decomposition [4, 5]. To obtain the sEMG envelope, the denoised signal was smoothed using a root-mean-square filter with a 250 ms window. All muscles exhibit a short latency between neural drive and generated force. This is referred to as a neuromechanical delay and has been investigated in the past for various muscles. For the diaphragm values of about 18 ms have been reported [6]. Ideally, the sEMG envelope would directly compensate for this effect, so we parameterized the filter lag to minimize the delay between electrical muscle activity and P_{mus}. This global filter lag was determined by maximizing the cross-correlation between the sEMG envelope and the P_{mus} waveform across all datasets. Finally, we applied simple heuristics to select the sEMG channel that is most informative for the automatic segmentation of inspirations as described in appendix B.1.

Appendix B: Criteria

B.1 sEMG signal criteria for automated detection

The properties of respiratory sEMG signals vary between patients, recordings, and channels. Various factors influence the amplitude and other features, such as the recorded muscles, electrode positions, and characteristics of tissue between electrodes and the target muscle. Furthermore, the patient activity, to be represented by sEMG, depends on the patient's condition, which is influenced by the disease state, medication and sedation, and the degree of ventilation. Another impact to be considered is crosstalk by other muscles, specifically expiratory crosstalk. As a result, automated sEMG detection is not always possible and not always useful. Specifically, the signal should have a minimum signal strength and should show mainly inspiratory activity.

To investigate these requirements, we characterized each sEMG signal using the signal-to-noise ratio (SNR) and the inspiratory-to-expiratory amplitude ratio (IEAR). The SNR is calculated according to Graßhoff *et al.* [7] and is a measure of

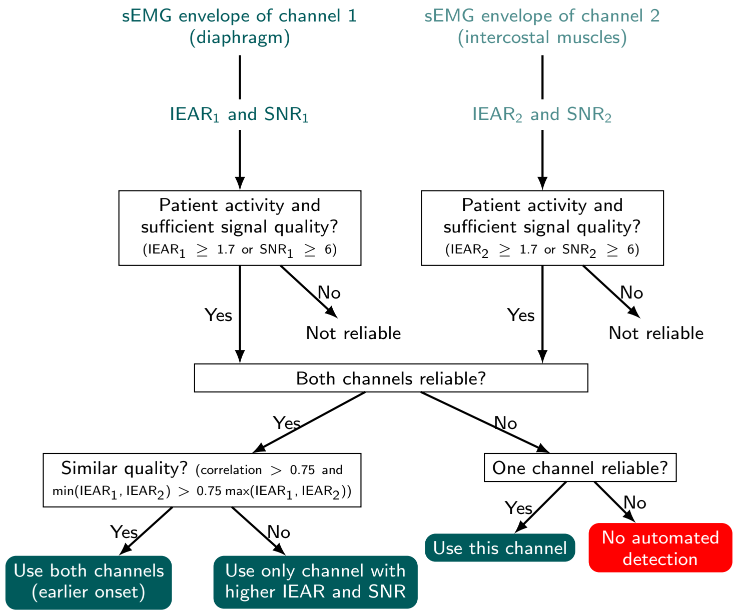

Figure B.1: Visualization of sEMG signal criteria in the form of a decision tree based on SNR (signal-to-noise ratio) and IEAR (inspiration-to-expiration ratio). If both channels show patient activity and are of similar and sufficient quality, both are automatically segmented, and the earlier onset (breath-by-breath) is used. In case one channel is not reliable or has a much lower signal quality, only one channel is used. If no channel shows patient activity or the signal quality is too low, no automated segmentation is applied.

the signal strength, which relates the signal amplitude to the background noise amplitude. The second ratio characterizes the level of expiratory crosstalk contamination and is calculated as follows. The sEMG signal is segmented using the airflow $\dot{V}$ into inspiratory (positive $\dot{V}$) and expiratory phases (negative $\dot{V}$). To remove the transitions between inspiration and expiration and vice versa, each segment's size is further decreased by the first and last quarters. Next, in each segment, the amplitude is estimated using the 0.9-quartile. Subsequently, all inspiratory segments are condensed by their 0.75-quartile. The same applies to all expiration phases. The final IEAR is calculated from the relation of obtained inspiratory to expiratory signal amplitude. Using quartiles ensures robustness to outliers. Additionally, as the IEAR is calculated on multiple breathing cycles (minimum 15), we assumed that the effect of auto and ineffective triggers can be neglected.

Figure B.1 visualizes the sEMG signal criteria based on SNR and IEAR. The two ratios were calculated for each recording, and both measured sEMG channels. First, both channels were evaluated individually. Second, if both channels show patient activity and no crosstalk, they are compared. If they show similar values for IEAR and are correlated, both are used (example in fig. B.2d). Otherwise, only the better channel is automatically segmented (example in fig. B.2c). The same applies if only one channel is reliable (example in fig. B.2b). No automated detection is performed if no channel shows patient activity and sufficient quality (example in fig. B.2a).

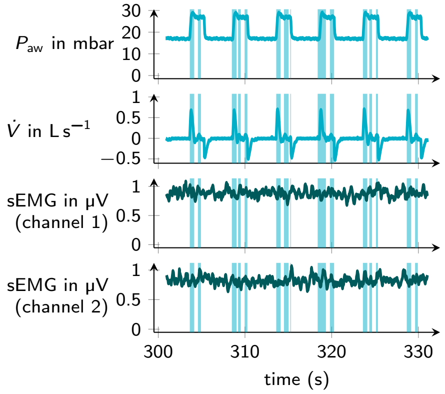

(a) No automated detection ($SNR_1 = 1.9$, $IEAR_1 = 1.0$, $SNR_2 = 1.9$ and $IEAR_2 = 0.9$)

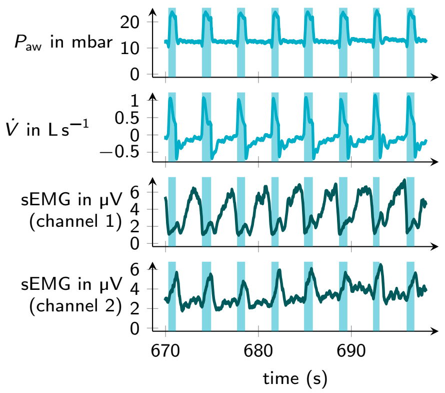

(b) Only channel 2 is reliable ($SNR_1 = 3.3$, $IEAR_1 = 0.3$, $SNR_2 = 2.8$ and $IEAR_2 = 1.7$)

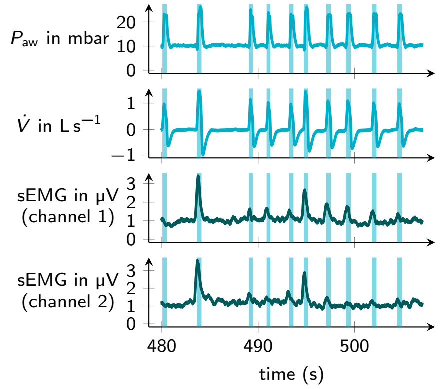

(c) Both channels are considered reliable but only channel 1 is used, because it shows higher signal amplitudes ($SNR_1 = 3.1$, $IEAR_1 = 7.1$, $SNR_2 = 3.1$ and $IEAR_2 = 4.3$)

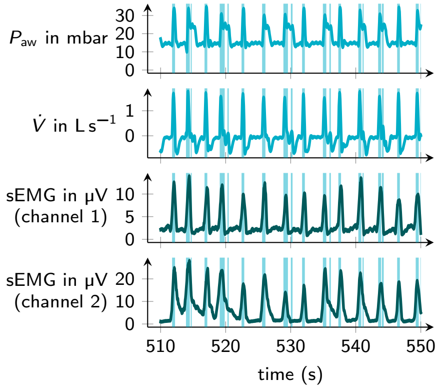

(d) Both channels are segmented and the earlier onset is used ($SNR_1 = 3.0$, $IEAR_1 = 5.4$, $SNR_2 = 12.0$ and $IEAR_2 = 4.4$)

Figure B.2: Examples for SNR (signal-to-noise ratio) and IEAR (inspiration-to-expiration signal ratio and the automated decision based on the signal criteria in fig. B.1. Each subfigure shows (top to bottom) the P_{aw} and $\dot{V}$ waveforms and both sEMG envelopes, where channel 1 corresponds the measurements of the diaphragm and channel 2 records the auxiliary muscles.

B.2 Ventilation mode criteria for PVI characterization

In this study, the patient-ventilator asynchrony was analyzed automatically, based on segmentations of the inspiratory patient effort in sEMG measurements and the segmentation of the ventilator support in the airway P_{aw}. The study analysis considers trigger asynchronies. Therefore, only recordings are included in the analysis, where patients were ventilated in pressure support modes, with well-distinguishable inspiration and expiration phases. We especially excluded CPAP and CPAP with tube compensation settings.

Appendix C: sEMG detection algorithms

Respiratory sEMG signals contain unspecific background noise. This affects the envelope in the form of a varying offset. Prior to activity detection, the offset of the envelope was removed. For this purpose, the procedure described by Graßhoff *et al.* [7] was used.

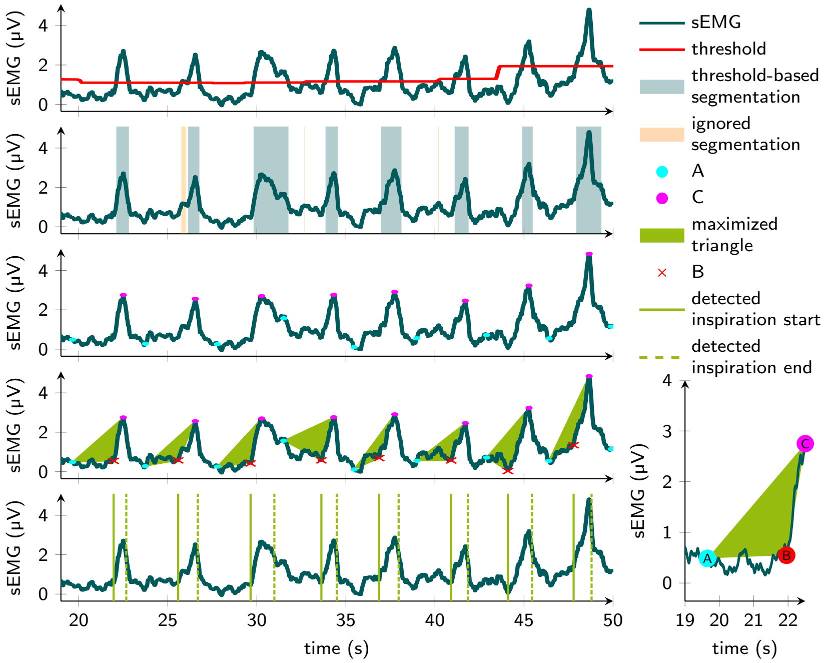

Figure C.1: Illustration of the substeps of the triangle algorithm on an example with low SNR. The first plot shows the amplitude-based threshold, leading to the early segmentation in the second plot. Yellow segments are excluded, because they are too short. For applying the triangle maximization procedure [8], points (A) during expiration and (C) during inspiration are determined. The third triangle corner (B) marks the beginning of inspiration. The first inspiratory activity is displayed enlarged on the right side.

C.1 Triangle algorithm

The first sEMG detection algorithm consists of two phases. The first step segments the signal using a coarse but slowly adapting threshold. The threshold is calculated as 40 % of the maximum amplitude of the sEMG envelope within a sliding 10 s window. Subsequently, segments shorter than 300 ms are removed, and if the gap between two segments is shorter than 400 ms, the gap is closed, and both segments are combined into one. Each segment is assigned to one inspiratory process. The exact onset of inspiration is then calculated, similar to the procedure proposed by Garcia-Castellote *et al.* [8]. A visualization of this method is presented in fig. C.1. First, points (A) and (C) are determined for each segment. (C) is positioned on the maximum of the segmented area, and point (A) is set to the beginning of the previous expiratory phase. For this, 30 % of the distance between (C) and the maximum of the previous segment is assumed. A third point, (B), is now placed on the sEMG envelope between (A) and (C) so that the area of the spanned triangle is maximized. Point (B) is used as the start of inspiration. This point can also lie outside the earlier segmentation.

C.2 Adaptive thresholding algorithm

The second algorithm is inspired by the defragmentation method proposed by Sinderby *et al.* [9] for EAdi, which was modified in this work to better account for the characteristics of sEMG. Briefly, a highly sensitive, adaptive threshold was used

to detect the onsets of inspirations, and then simple defragmentation rules were applied to reject false positives or merge adjacent breaths.

The adaptive threshold is calculated as 10 % above the median sEMG envelope in a 3.5 s moving window. The segmentation is implemented using the state diagram in fig. C.2a, which runs over the data from left to right. An onset is detected when a sample exceeds the adaptive threshold and has a positive slope (i.e., it is larger than the previous sample). When the signal drops below the threshold (before reaching the end-of-inspiration criterion) the breath is rejected. When the end-of-inspiration criterion is reached (signal drops below 70 % of the maximum), the current breath is rejected if its too short (< 300 ms) and it is merged with the previous breath when there is only a small gap between the two (< 350 ms between the previous and current breath). If the rejection/merge criteria are not met, the current breath is considered to be valid, and the start/end points are stored. The algorithm then returns to its initial state and repeats all of the detection steps described above.

Author details

References

1. Graßhoff, J., Petersen, E., Eger, M., Bellani, G., Rostalski, P.: A template subtraction method for the removal of cardiogenic oscillations on esophageal pressure signals. In: 2017 39th Annual International Conference of the IEEE Engineering in Medicine and Biology Society (EMBC), pp. 2235–2238. IEEE, ??? (2017). doi:10.1109/embc.2017.8037299
2. Baldwin, E.d., Cournand, A., Richards Jr, D.W.: Pulmonary insufficiency: I. physiological classification, clinical methods of analysis, standard values in normal subjects. Medicine **27**(3), 243–278 (1948)
3. Mauri, T., , Yoshida, T., Bellani, G., Goligher, E.C., Carteaux, G., Rittayamai, N., Mojoli, F., Chiumello, D., Piquilloud, L., Grasso, S., Jubran, A., Laghi, F., Magder, S., Pesenti, A., Loring, S., Gattinoni, L., Talmor, D., Blanch, L., Amato, M., Chen, L., Brochard, L., Mancebo, J.: Esophageal and transpulmonary pressure in the clinical setting: meaning, usefulness and perspectives. Intensive Care Medicine **42**(9), 1360–1373 (2016). doi:10.1007/s00134-016-4400-x
4. Pan, J., Tompkins, W.J.: A real-time QRS detection algorithm. IEEE Transactions on Biomedical Engineering **BME-32**(3), 230–236 (1985). doi:10.1109/tbme.1985.325532
5. Petersen, E., Sauer, J., Graßhoff, J., Rostalski, P.: Removing cardiac artifacts from single-channel respiratory electromyograms. IEEE Access **8**, 30905–30917 (2020). doi:10.1109/access.2020.2972731
6. Bellemare, F., Bigland-Ritchie, B., Woods, J.J.: Contractile properties of the human diaphragm in vivo. Journal of Applied Physiology **61**(3), 1153–1161 (1986). doi:10.1152/jappl.1986.61.3.1153
7. Graßhoff, J., Petersen, E., Farquharson, F., Kustermann, M., Kabitz, H.-J., Rostalski, P., Walterspacher, S.: Surface EMG-based quantification of inspiratory effort: a quantitative comparison with Pes. Critical Care **25**(1) (2021). doi:10.1186/s13054-021-03833-w
8. Garcia-Castellote, D., Torres, A., Estrada, L., Sarlabous, L., Jané, R.: Evaluation of indirect measures of neural inspiratory time from invasive and noninvasive recordings of respiratory activity. In: 2017 39th Annual International Conference of the IEEE Engineering in Medicine and Biology Society (EMBC), pp. 341–344 (2017). doi:10.1109/EMBC.2017.8036832
9. Sinderby, C., Liu, S., Colombo, D., Camarotta, G., Slutsky, A.S., Navalesi, P., Beck, J.: An automated and standardized neural index to quantify patient-ventilator interaction. Critical Care **17**(R239) (2013). doi:10.1186/cc13063

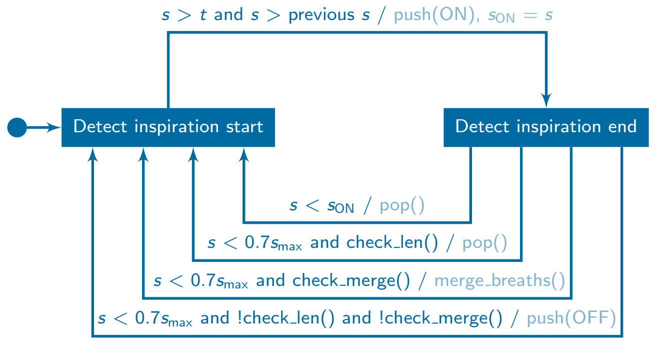

variables

s	: sEMG amplitude of current sample
t	: threshold
s_{ON}	: sEMG amplitude at inspiration start
s_{max}	: maximum sEMG amplitude during current inspiration
push()	: add sample to stack
pop()	: remove the last sample from stack
check_len()	: check minimum duration of inspiration
check_merge()	: check maximum distance of inspirations
merge_breaths()	: merge this and previous inspiration

(a) The state diagram shows a Mealy machine, where each edge is labeled with input / output.

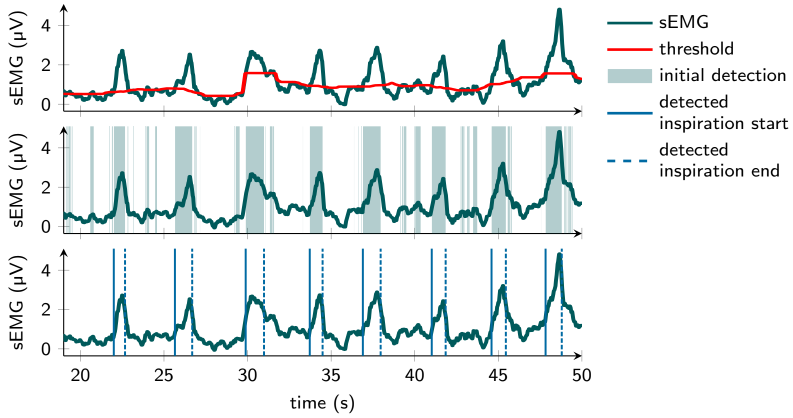

(b) Visualization of the detection procedure using the triangle algorithm on the same example as in fig. C.1. In the first plot, the adaptive threshold is given in red. The second row shows the intermediate inspiration starts and ends. In the bottom row, only accepted detections are displayed.

Figure C.2: Overview on detection procedure of the adaptive thresholding algorithm.
