# Supplementary material for: Automated characterization of patient–ventilator interaction using surface electromyography
Source: Ann Intensive Care. 2024 Feb 26;14:32. doi: 10.1186/s13613-024-01259-5 (PMC10897101; doi:10.1186/s13613-024-01259-5)
Supplement: Supplementary file 2 — Additional file 2: Figure S1. Overview on number of recordings per patient. Table S1. Number of annotated/detected breaths for each patient and recording. Figure S2. Detection validation against Pes reference. Table S2. Numerical results for sEMG detection performance. Table S3. Numerical results for PVI classification evaluation. Figure S3. Detection validation against sEMG reference. [file 13613_2024_1259_MOESM2_ESM.pdf]

Number of recordings, annotations and detected breaths

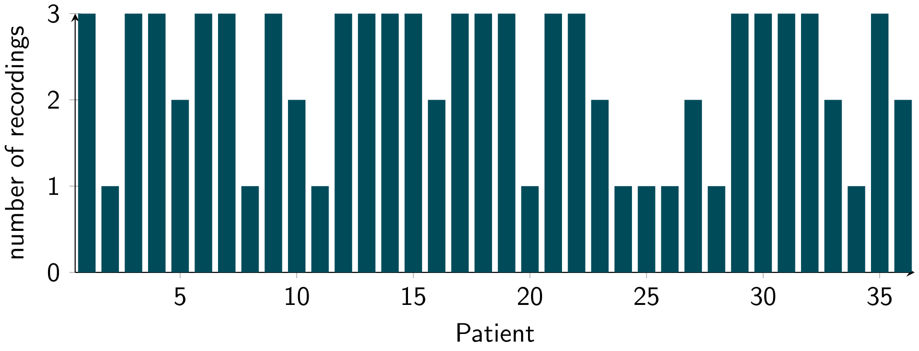

Figure S1 Overview on number of recordings per patient.

Table S1 Number of annotated/detected breaths for each patient and recording.

Patient (Recording)	P_{es} Expert 1	P_{es} Expert 2	P_{es} Valid (%)	sEMG (costal margin) Triangle algorithm	sEMG (costal margin) Adaptive thresholding algorithm	sEMG (parasternal) Triangle algorithm	sEMG (parasternal) Adaptive thresholding algorithm	earliest sEMG (diaphragm in %) Triangle algorithm	earliest sEMG (diaphragm in %) Adaptive thresholding algorithm
1 (2)	103	107	103 (96)	101	142	99	140	101 (100)	142 (100)
1 (3)	101	103	100 (96)	101	143	99	123	101 (100)	143 (100)
3 (2)	99	119	96 (81)	144	148	146	148	144 (100)	148 (100)
3 (3)	101	101	101 (100)	99	101	103	103	103 (19)	104 (81)
4 (3)	0	0	0 (0)	0	0	114	124	114 (0)	124 (0)
6 (2)	110	109	59 (52)	0	0	122	161	122 (0)	161 (0)
9 (1)	99	93	75 (71)	0	0	232	272	232 (0)	272 (0)
9 (2)	104	98	80 (72)	89	111	81	114	89 (100)	111 (100)
9 (3)	95	102	89 (85)	87	132	80	126	87 (100)	132 (100)
10 (1)	43	40	35 (74)	78	85	53	81	53 (0)	81 (0)
10 (2)	105	111	101 (90)	0	0	102	115	102 (0)	115 (0)
12 (2)	172	176	146 (79)	189	201	190	200	190 (2)	200 (2)
12 (3)	114	117	101 (85)	127	133	127	137	127 (100)	133 (100)
13 (2)	1	6	0 (0)	0	0	26	28	26 (0)	28 (0)
13 (3)	107	109	102 (93)	113	162	133	140	113 (100)	162 (100)
14 (3)	101	104	101 (97)	182	161	120	108	120 (1)	108 (1)
15 (2)	129	131	125 (95)	0	0	0	0	0	0
15 (3)	0	0	0 (0)	120	132	112	132	120 (100)	132 (100)
16 (2)	106	111	106 (95)	108	110	108	112	109 (20)	114 (52)
17 (2)	118	117	117 (99)	121	131	123	127	121 (100)	131 (100)
17 (3)	118	117	117 (99)	118	118	112	115	118 (45)	118 (47)
18 (2)	73	72	72 (99)	0	0	76	77	76 (0)	77 (0)
18 (3)	114	123	105 (85)	0	0	137	122	137 (0)	122 (0)
19 (2)	122	122	122 (100)	120	124	121	125	121 (6)	125 (3)
21 (1)	109	106	104 (95)	0	0	108	113	108 (0)	113 (0)
22 (2)	106	104	81 (76)	111	152	103	124	111 (100)	152 (100)
22 (3)	99	101	92 (91)	142	142	99	102	99 (1)	102 (1)
29 (2)	114	114	112 (98)	129	132	123	133	123 (0)	133 (2)
29 (3)	97	97	96 (98)	85	95	0	0	85 (100)	95 (100)
30 (2)	116	117	113 (96)	0	0	0	0	0	0
30 (3)	106	107	105 (98)	0	0	107	159	107 (0)	159 (0)
32 (3)	104	104	104 (100)	105	107	97	103	105 (100)	107 (100)
33 (1)	100	101	100 (99)	100	104	100	106	100 (7)	109 (48)
33 (2)	106	106	106 (100)	0	0	106	108	106 (0)	108 (0)
35 (1)	90	169	90 (53)	80	143	90	155	80 (100)	143 (100)
35 (3)	101	100	66 (65)	157	125	0	0	157 (100)	125 (100)
36 (1)	103	103	83 (81)	103	114	103	110	103 (19)	121 (29)

For each recording, the number of annotated breaths in P_{es} by each expert individually is given. Additionally, the number of breaths, where both experts agreed, is given (valid breaths). In this work, an agreement is assumed if both experts have detected the same inspiratory effort and the distance between both annotated onsets is less than 250 ms. Furthermore, the table shows the number of automatically detected breaths in both sEMG channels by both algorithms. A number of zero means that signal quality criteria (e.g., based on the SNR) had not been fulfilled, thus no automated detection was conducted. Next, both sEMG channels are fused by always using the earlier onset if an inspiration is detected in both channels. The number in brackets provides the portion of breaths that were detected earlier in the diaphragm. Datasets where neither any expert has observed patient effort in P_{es} nor the automated detection was possible are left out.

Detection validation against P_{es}

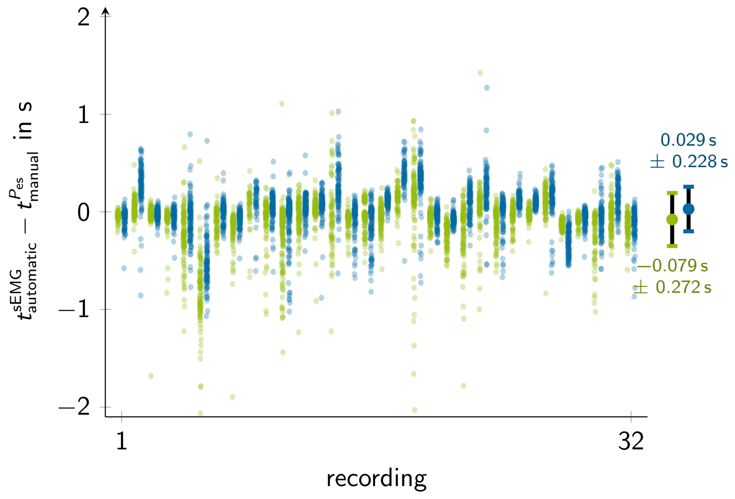

Figure S2 Detection validation against P_{es} reference. The validity of both approaches is examined by comparing automatically detected inspirations in the sEMG $t^{\mathrm{sEMG}}_{\mathrm{automatic}}$ and manual annotations in the esophageal pressure $t^{P_{\mathrm{es}}}_{\mathrm{manual}}$. This plot shows the temporal difference of correctly detected onsets of inspiration. For both algorithms, the mean and standard deviation over all inspirations are displayed on the right side.

Table S2 Numerical results for sEMG detection performance.

Metric	Triangle algorithm	Adaptive thresholding algorithm
**Sensitivity**	0.97 (0.91-0.99)	1.00 (0.98-1.00)
**PPV**	0.94 (0.85-1.00)	0.87 (0.72-0.93)
**Detection error**	-0.079 ± 0.272	0.029 ± 0.228
**Absolute detection error**	0.156 ± 0.240	0.166 ± 0.164

For sensitivity and PPV (positive predictive value), the median and interquartile range across all recordings are given. The mean and standard deviation of the detection deviation $t_{\text{automatic}} - t_{\text{manual}}$ and absolute detection deviation $|t_{\text{automatic}} - t_{\text{manual}}|$ is calculated over all correctly detected patients efforts and is given in seconds.

Table S3 Numerical results for PVI classification evaluation.

	**PVI classification** Reference based on $t_{\text{manual}}^{P_{\text{es}}}$					
**Class**	**Sensitivity**		**Specificity**		**PPV**	
	Triangle algorithm	Adaptive thresholding algorithm	Triangle algorithm	Adaptive thresholding algorithm	Triangle algorithm	Adaptive thresholding algorithm
**Synchronous trigger**	0.86 (0.67 to 0.93)	0.93 (0.89 to 0.99)	0.95 (0.78 to 1.00)	0.87 (0.70 to 1.00)	0.97 (0.87 to 1.00)	0.96 (0.83 to 1.00)
**Delayed trigger**	1.00 (0.46 to 1.00)	1.00 (0.15 to 1.00)	0.91 (0.83 to 0.98)	0.97 (0.94 to 1.00)	0.08 (0.00 to 0.77)	0.16 (0.00 to 1.00)
**Auto trigger**	1.00 (0.64 to 1.00)	0.81 (0.30 to 1.00)	0.99 (0.93 to 1.00)	1.00 (0.99 to 1.00)	0.62 (0.00 to 1.00)	1.00 (0.50 to 1.00)
**Ineffective trigger**	1.00 (0.98 to 1.00)	1.00 (1.00 to 1.00)	0.99 (0.92 to 1.00)	0.91 (0.81 to 0.96)	0.72 (0.12 to 1.00)	0.11 (0.00 to 0.34)
**Double trigger**	1.00 (1.00 to 1.00)	1.00 (1.00 to 1.00)	1.00 (1.00 to 1.00)	1.00 (1.00 to 1.00)	1.00 (0.95 to 1.00)	1.00 (1.00 to 1.00)
**Double effort**	1.00 (1.00 to 1.00)	1.00 (1.00 to 1.00)	1.00 (1.00 to 1.00)	1.00 (1.00 to 1.00)	1.00 (1.00 to 1.00)	1.00 (0.85 to 1.00)
**Total**	0.87 (0.79 to 0.98)	0.85 (0.76 to 0.97)	0.94 (0.91 to 0.97)	0.94 (0.91 to 0.96)	0.67 (0.54 to 0.83)	0.67 (0.52 to 0.79)
**Total (weighted)**	0.81 (0.66 to 0.90)	0.87 (0.75 to 0.95)	0.95 (0.84 to 1.00)	0.94 (0.78 to 1.00)	0.93 (0.82 to 0.98)	0.92 (0.82 to 0.98)

For each PVI class, the sensitivity, specificity, and PPV (positive predictive value) are calculated per recording. This figure shows the median value and the interquartile range across recordings. Reference for the sEMG-based PVI classification is the manual annotation of inspiratory efforts in the esophageal pressure by two experts.

Detection validation against manual sEMG reference

To assess the performance of the automated approach, the detected patient efforts in sEMG were also compared against expert annotations in sEMG. In the costal margin and parasternal sEMG channels, each expert marked the onset of inspiration based on the visible electrical activity in the signal.

The results in fig. S3 indicate that the triangle algorithm was more precise ($p = 0.001$), whereas the adaptive thresholding algorithm was more sensitive ($p = 0.002$). Both methods showed high median positive predictive value ≥ 0.92 and high median sensitivity ≥ 0.98. Among the correctly detected inspirations the detection deviation $t^{\mathrm{sEMG}}_{\mathrm{automatic}} - t^{\mathrm{sEMG}}_{\mathrm{manual}}$ was (-0.05 ± 0.22) s for the triangle algorithm and (0.06 ± 0.17) s for the adaptive thresholding algorithm. This means, on average, the first algorithm detected inspiratory activity earlier and the second one later than experts in the same signal.

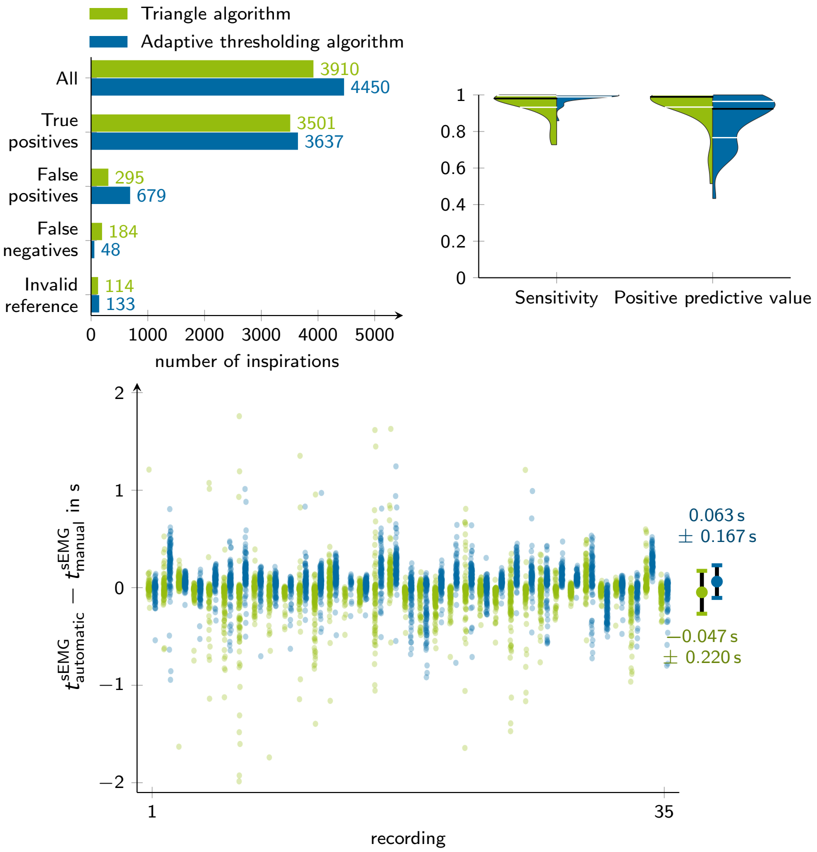

Figure S3 Detection validation against sEMG reference. The performance of both algorithms is examined by comparing automatically detected inspirations in the sEMG and manual annotations in the sEMG. The upper left plot overviews the total number of correctly and incorrectly detected patient efforts. The last entry shows how many of the detected breaths were assigned to an uncertain reference where the experts disagreed. The upper right plot shows binary metrics to evaluate the detection performance. For sensitivity and positive predictive value, the distribution over patients and recordings is given. Black lines denote the median value, and white lines visualize the interquartile range. This lower plot shows the temporal difference of correctly detected onsets of inspiration. For both algorithms, the mean and standard deviation over all inspirations are displayed on the right side.
